# An Immunological Axis Involving Interleukin 1β and Leucine-Rich-α2-Glycoprotein Reflects Therapeutic Response of Children with Kawasaki Disease: Implications from the KAWAKINRA Trial

**DOI:** 10.1007/s10875-022-01301-w

**Published:** 2022-06-14

**Authors:** Christoph Kessel, Isabelle Koné-Paut, Stéphanie Tellier, Alexandre Belot, Katja Masjosthusmann, Helmut Wittkowski, Sabrina Fuehner, Linda Rossi-Semerano, Perrine Dusser, Isabelle Marie, Nadja Boukhedouni, Helène Agostini, Céline Piedvache, Dirk Foell

**Affiliations:** 1 Department of Pediatric Rheumatology and Immunology, University Children’s Hospital Muenster, Domagkstr. 3, 48149 Muenster, Germany; 2 Division of Pediatric Rheumatology and CEREMAIA, Bicêtre Hospital, APHP, University of Paris Saclay, Le Kremlin-Bicêtre, France; 3 Department of Pediatrics, Divisions of Nephrology, Rheumatology and Internal Medicine, University of Toulouse, Toulouse, France; 4 Departments of Pediatrics, Division of Rheumatology, Dermatology and Nephrology, University of Lyon, Lyon, France; 5 Department of General Pediatrics, University Children’s Hospital Muenster, Muenster, Germany; 6 Clinical Research Unit Paris Saclay, APHP, Bicêtre Hospital, Paris Saclay, Le Kremlin-Bicêtre, France

**Keywords:** Kawasaki disease, Recombinant IL-1 receptor antagonist, Anakinra, Serum biomarkers

## Abstract

**Purpose:**

A recent phase II open-label study of the interleukin 1 (IL-1) receptor antagonist (IL-1Ra) anakinra in treating IVIG-resistant Kawasaki disease (KD) patients reported promising results. Here, we aimed to characterize the immunological impact of IL-1 blockade in this unique study population.

**Methods:**

Patients’ and control sera and supernatants of cells (whole blood, neutrophils, coronary artery endothelial cells) stimulated with recombinant IL-1β were analyzed for single or multiple marker (*n* = 22) expression by ELISA or multiplexed bead array assay. Data were analyzed using unsupervised hierarchical clustering, multiple correlation, and multi-comparison statistics and were compared to retrospective analyses of KD transcriptomics.

**Results:**

Inflammation in IVIG-resistant KD (*n* = 16) is hallmarked by over-expression of innate immune mediators (particularly IL-6 > CXCL10 > S100A12 > IL-1Ra). Those as well as levels of immune or endothelial cell activation markers (sICAM-1, sVCAM-1) declined most significantly in course of anakinra treatment. Prior as well as following IL-1R blockade, over-expression of leucine-rich-α2-glycoprotein 1 (LRG1) associated best with remnant inflammatory activity and the necessity to escalate anakinra dosage and separated inflammatory KD patients from sJIA-MAS (*n* = 13) and MIS-C (*n* = 4). Protein as well as retrospective gene expression analyses indicated tight association of LRG1 with IL-1β signaling and neutrophilia, while particularly neutrophil stimulation with recombinant IL-1β resulted in concentration-dependent LRG1 release.

**Conclusion:**

Our study identifies LRG1 as known trigger of endothelial activation and cardiac re-modeling to associate with IL-1β signaling in KD. Besides a potential patho-mechanistic implication of these findings, our data suggest blood leukocyte and neutrophil counts to best predict response to IL-1Ra treatment in IVIG-resistant KD.

**Supplementary Information:**

The online version contains supplementary material available at 10.1007/s10875-022-01301-w.

## Introduction

Kawasaki disease (KD) is an acute systemic vasculitis of unknown etiology that affects small-sized and medium-sized arteries of infants and children. It is the main cause of acquired heart disease during childhood in developed countries. A single infusion of 2 g/kg of intravenous immunoglobulins (IVIG) along with aspirin has reduced the frequency of coronary artery aneurysms (CAA) from 25 to 5%. However, 10–20% of patients are unresponsive to IVIG treatment and thus present with persisting fever and inflammation, and have a threefold increased risk of cardiac complications and death [1, 2].

Elevated levels of inflammatory cytokines such as tumor necrosis factor α (TNF) and IL-6 primed investigations of cytokine targeting strategies as rescue therapies in case of IVIG resistance. Yet, even though both therapeutic TNFα-neutralization [3] and IL-6R-blockade [4] either in addition to IVIG treatment [3] or as prospective pilot study in IVIG-resistant cases [4] contributed to improvement of some clinical and laboratory measures [3, 4], these approaches did not reduce treatment resistance [3]. Pilot study data regarding IL-6R-blockade even suggest an association of therapy with formation of new-onset CAA [4].

Apart from TNF and IL-6, compelling evidence gathered over recent years points to a major role of NLRP3 inflammasome activation and IL-1 family cytokines in KD. This finding is all the more intriguing as it highlights a significant pathophysiological overlap between KD and systemic juvenile idiopathic arthritis (SJIA, Still’s disease), in addition to some of the common clinical features already noted, notably the potential progression to macrophage activation syndrome (MAS) [5].

Historic data already demonstrated the prevalence of elevated IL-1 pathway gene signatures in KD [6, 7]. Furthermore, spontaneous IL-1β release from in vitro cultured peripheral blood mononuclear cells (PBMCs) obtained from KD patients and responsive to IVIG treatment has been shown [8]. Elevated circulating IL-1 and IL-18 serum levels separated acute KD children from febrile controls and decreased in course of convalescence [9]. NLRP3-associated gene expression might reflect KD disease activity, while polymorphisms in genes encoding proteins involved in regulating intracellular Ca^2+^ flux as a prominent NLRP3 inflammasome activator have been found to associate with KD [9]. Inflammatory activation of human coronary artery endothelial cells showed to be particularly sensitive to IL-1β released from human monocytes upon toll-like receptor 4 stimulation and subsequent inflammasome activation [10]. Similarly, disease development in the *Lactobacillus casei* cell wall extract (LCWE)-induced KD mouse model required IL-1 producing macrophages as well as MyD88-signaling in hematopoietic cells [11]. Mirroring an increased KD incidence in male patients, male mice subjected to the LCWE-induced KD mouse model also presented with a more severe IL-1β-dependent disease phenotype [12]. Animals subjected to this pre-clinical KD model were protected from disease when receiving either IL-1α or IL-1β neutralizing antibodies, but protection was most evident when treatments were combined [11, 13] or signaling through the IL-1 receptor was blocked using the recombinant IL-1 receptor antagonist (IL-1Ra) anakinra [12, 14].

Built on this collective evidence, several clinical trials aiming to evaluate the efficacy of IL-1 blocking therapies in KD have been initiated [15, 16]. Recently, results from the first open-label, phase IIa clinical trial (KAWAKINRA, Eudract Number: 2014–002,715-41, ClinicalTrials.gov NCT02390596) evaluating the safety of anakinra in IVIG-resistant KD patients have been published [17]. These data support the early use of anakinra in IVIG-refractory cases, demonstrating that it is quickly effective on KD symptoms, inflammatory parameters, and coronary artery dilations in most patients, with good safety [17]. Similarly, a second study assessing anakinra therapy in patients with acute KD and CAAs reported favorable safety and efficacy data [16].

In this follow-up study, we investigated the impact of IL-1 blockade on circulating serum markers among KAWAKINRA participants and identified leucine-rich-α2-glycoprotein 1 (LRG1) to consistently associate with remnant inflammatory activity and the necessity to escalate anakinra dosage in IVIG-resistant KD patients and to separate those from sJIA-MAS as well as MIS-C. Both protein and retrospective analyses of historic gene expression data indicated tight association of LRG1 with IL-1β signaling and neutrophilia, while in vitro neutrophil stimulation with recombinant IL-1β resulted in concentration-dependent LRG1 release.

## Materials and Methods

### Study Participants, Design, and Sample Collection

Study participants (Table 1), design, and outcome parameters have already been reported elsewhere [15]. Briefly, the KAWAKINRA trial (Eudract Number: 2014–002,715-41, ClinicalTrials.gov NCT02390596) enrolled sixteen IVIG-resistant KD patients (resistant to 1–3 cycles of IVIG) who received subcutaneous anakinra at a starting dosage of 2 or 4 mg/kg/day (depending on age and body weight), which was escalated every 24 h if patients would not become afebrile (> 38 °C). Patients received daily anakinra injection for a total duration of 14 days and the primary outcome parameter was abatement of fever. One patient (no. 15) was retrospectively diagnosed as systemic JIA complicated by macrophage activation syndrome and a second patient (no. 8) received only a single anakinra injection due to false initial dosage. For the purpose of the present study, patients’ serum was collected at screening visit (prior to anakinra), at study day 3 (following 3 anakinra injections) and at day 14 (end of anakinra treatment).

**Table 1:** Study population

	IVIG-resistant KD (KAWAKINRA study cohort [17]) *n* = 16	sJIA-MAS (*n* = 9)	MIS-C (*n* = 4)	Pediatric HC (*n* = 4)
Sex, no. (%)				
Female	2 (13)	5 (55)	1 (25)	2 (50)
Male	14 (87)	4 (45)	3 (75)	2 (50)
Age (years), median (range)	2.58 (0.25–6.9)	17.8 (8–19)	10 (8–15)	11 (7–15)
Clinical laboratory parameters, median (range)				
CRP, mg/dL	13.5 (2.5–40.3)^§^	11.8 (5–25.7)	26.3 (11.8–32.7)	n.d
Ferritin, µg/L	n.d	3385 (1476–25,977)	2749 (1039–4195)	n.d
Hemoglobin, d/dL	9.2 (7.6–11.9)^§^	11.1 (9.6–11.6)	10.5 (7.8–11.9)	n.d
Leukocytes/mm^3^	15,335 (6999–30,550)^§^	7080 (2700–34,300)	24,590 (17,750–30,860)	n.d
Neutrophils/mm^3^	10,375 (3600–28,530)^§^	4270 (400–8800)	9863 (6380–14,470)	n.d
Thrombocytes × 10^9^/L	496 (194–879)^§^	158 (13–446)	117.9 (102–776)	n.d
Medication				
Naive	0/16	1/9	0/4	4/4
IVIG	16/16	0/9	4/4	0/4
Anakinra	16/16	4/9	0/4	0/4
Steroids	3/16	8/9	4/4	0/4
Tocilizumab	0/16	2/9	0/4	0/4
CSA	0/16	2/9	0/4	0/4

*CRP* C-reactive protein; *HC* healthy control; *IVIG* intravenous immunoglobulin; *KD* Kawasaki disease; *MIS-C* multi-system inflammatory syndrome in children

^§^Clinical laboratory values at screening visit

Sera of healthy pediatric controls (*n* = 4), patients with macrophage activation syndrome (MAS) associated with systemic juvenile idiopathic arthritis (sJIA; *n* = 8), and MIS-C patients (*n* = 4) were collected at University Children’s Hospital Muenster, Germany and have in part already been reported elsewhere (Table 1) [18]. Serum collection was approved by the local (University Hospital Muenster: 2015–670-f-S) ethical committee and parents or caregivers signed written informed consent. Whole blood and neutrophils were isolated from healthy adult volunteers.

### Cell Stimulations and Single or Multiplexed Analyses of Supernatants and Serum Specimens

Fresh human whole blood, primary polymorphonuclear leukocytes (PMNs), or human coronary artery endothelial cells (HCAECs) were stimulated in vitro and supernatants as well as sera of KAWAKINRA study participants and healthy controls were analyzed by multiplexed bead array assay (Luminex) and single-marker ELISA (S100A12) as detailed in supplemental methods.

### Data Analysis

Serum marker data were analyzed for unsupervised clustering using correlation distance and ward.D linkage by the pheatmap R package and RStudio (RStudio Team (2015). RStudio: Integrated Development for R. RStudio, Inc., Boston, MA http://www.rstudio.com/). Multiple Spearman rank correlation analyses of serum analytes were performed and plotted using the GraphPad Prism software (version 8.0 for Mac OS X, GraphPad Software, La Jolla, CA, USA).

Data of individual serum markers were analyzed for normality distribution by D’Agostino and Pearson normality test using the GraphPad Prism software. The large majority of data did not pass this test and data were therefore subjected to non-parametric Mann–Whitney *U* or multi-comparison analyses by Kruskal–Wallis followed by Dunn’s multiple comparison test or Friedmann’s multiple comparison test for analyses of paired samples (GraphPad Prism v8.0). Receiver operating characteristic (ROC) curve analyses were performed using the GraphPad Prism software.

In clustering or multiple correlation analyses of serum markers, we widely used raw mean fluorescence intensity (MFI) values instead of absolute concentrations as this offers the advantage to analyze data without the restrains of lower or upper limit of detection [19].

Retrospective analyses of KD transcriptomic data was performed on GSE63881 [7]. Expression based on *Z* score of raw values normalized by log10 transformation was analyzed for *LRG1* (ILMN_1805228), *IL1R1* (ILMN_1810584), *IL1B* (ILMN_1775501), *S100A12* (ILMN_1748915), *IL6* (ILMN_1699651), *IL6RA* (ILMN_1696394), and *IL6RB* (ILMN_1754753) using the GraphPad Prism software.

## Results

### Characterization of IVIG-Resistant Systemic Inflammation in KD Patients

**Fig. 1:**
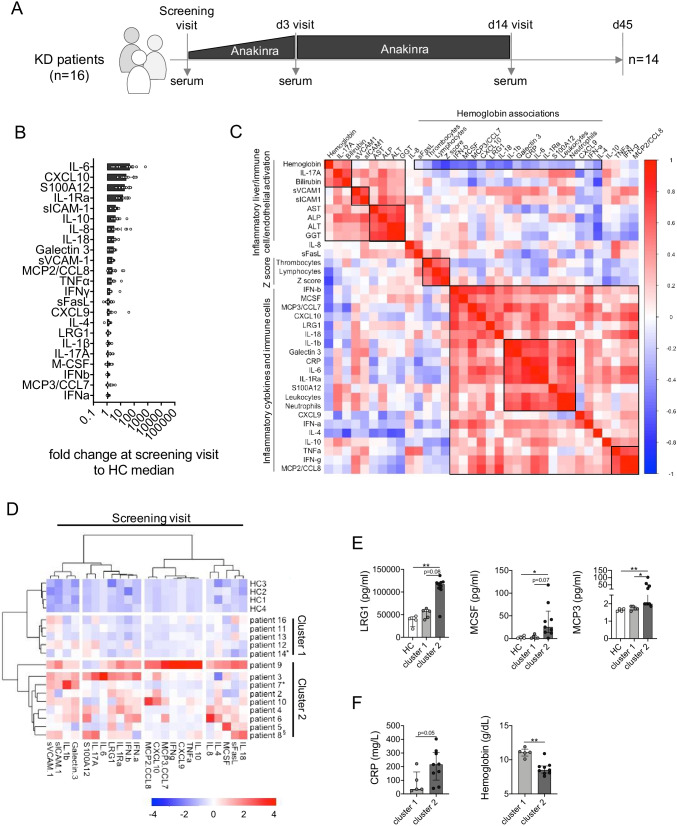
IVIG-resistant systemic inflammation in KD patients is hallmarked by specific innate immune mediators. **A** Schematic representation of the KAWAKINRA study protocol indicating sampling at screening visit (prior to anakinra). **B** Fold change (on MFI level) of blood biomarkers assessed in the present study (*n* = 23) over pediatric healthy control median. **C** Multiple correlation analyses of blood biomarkers, clinical markers of inflammation, and circulating cell counts. Black squares are used to highlight prominent clusters of association, and color coding reflects Spearman correlation coefficient. **D** Ward’s unsupervised hierarchical clustering of serum biomarker levels quantified at screening visit. Color coding indicates *Z* score. **E**, **F** Serum biomarkers (**E**) and inflammatory parameters (**F**) reflecting the clustering of patients. Data were analyzed by Kruskal–Wallis followed by Dunn’s multiple comparison (**E**) or Mann–Whitney *U* test (**F**). * = *p* < 0.05; ** = *p* < 0.01. ^§^Patient 8 received steroids prior to anakinra; *patients 7 and 14 received two cycles of IVIG prior to anakinra. No screening visit serum sample was available for patient 1

At screening visit (prior to anakinra, Fig. 1A), circulating levels of IL-6 (median fold change vs. HC: 48.25), CXCL10 (26.67), S100A12 (20.27), and IL-1Ra (15.18) were most prominently upregulated compared to healthy controls (Fig. 1B). Multiple correlation analyses of blood biomarkers, cell counts, clinical routine inflammatory parameters, and *Z* scores have been performed to provide an overview of the inflammatory status-quo of IVIG-resistant KD patients prior to anakinra treatment. This identifies numerous associations between inflammatory cytokines and immune cells, with two prominent clusters of strong positive correlation comprising IL-1b, IL-1RA, IL-6, S100A12, Galectin 3, CRP, and particularly neutrophils as well as TNF, IFN-γ, and MCP-2 (Fig. 1C). Coronary artery *Z* score at screening visit was predominantly associated with lymphocyte and thrombocyte counts as well as hemoglobin concentration rather than inflammatory mediators. Markers of liver function (bilirubin, AST, ALP, ALT, GGT) revealed pronounced positive correlation with cellular adhesion molecules (sICAM-1, sVCAM-1) as well as IL-17A. In contrast to many other inflammatory parameters, both IL-17A and bilirubin were positively associated with hemoglobin concentrations (Fig. 1C). Particularly the latter association may eventually account for IVIG-related hemolysis [20], which we did not investigate in more detail.

Unsupervised hierarchical clustering according to correlation distance and Ward’s linkage of serum marker data obtained from samples at screening visit (prior to anakinra) highlights differing levels of systemic inflammatory activity at study entry (Fig. 1D). Blood biomarkers in patients’ samples constituting cluster 1 were hardly elevated over those in healthy controls, whereas cluster 2 comprised patients with in part substantial elevation of serum markers (Fig. 1D). From this as well as all subsequent analyses, we excluded patient 15, who was retrospectively diagnosed with sJIA-associated MAS (sJIA-MAS) and did already present with strongly elevated serum levels of both S100A12 and IL-18 as hallmarks of sJIA-MAS [21] at screening visit (Figure S1A). Of note, all study participants received only one IVIG infusion prior to anakinra, except for patients 7 and 14 (two IVIG infusions) and patient 15 (three IVIG infusions). No serum sample at screening visit was available for patient 1.

Upon single-marker analyses, we observed absolute levels of LRG1, M-CSF, and MCP3 to best separate patients in cluster 2 from cluster 1 and healthy controls (Fig. 1E). At MFI level, IL-1β and IFN-β were also significantly elevated among patients in cluster 2 but overall levels were too low to allow for robust conversion to absolute concentration (Figure S1B). While many other of the quantified blood biomarkers were elevated among individuals in cluster 2 over those in cluster 1 and healthy controls, those differences in expression remained far below significance level (Figure S1C). Furthermore, concentrations of CRP and hemoglobin as classical markers of inflammation indicated higher inflammatory activity among patients in cluster 2 (Fig. 1F). This was not reflected by blood cell counts (Figure S1D).

As already obvious from correlation analyses of multiple laboratory and clinical markers at screening visit (Fig. 1C), at expression level (MFI), we observed significant association between LRG1 and M-CSF (*r*_*s*_ = 0.57, *p* = 0.038) or MCP3 (*r*_*s*_ = 0.60, *p* = 0.025) and IFN-β (*r*_*s*_ = 0.76, *p* = 0.003) as well as IL-1β by trend (*r*_*s*_ = 0.52, *p* = 0.058). Expression of IL-1β—as the treatment target in this study—was further significantly associated with IL-1Ra (*r*_*s*_ = 0.76, *p* = 0.002), M-CSF (*r*_*s*_ = 0.55, *p* = 0.044), MCP3 (*r*_*s*_ = 0.80, *p* = 0.001), and IFN-β levels (*r*_*s*_ = 0.71, *p* = 0.006), as well as CRP (*r*_*s*_ = 0.59, *p* = 0.028) and hemoglobin concentrations (*r*_*s*_ =  − 0.59, *p* = 0.029).

### Association of Anakinra Treatment and Dosage with Blood Biomarker Levels

**Fig. 2:**
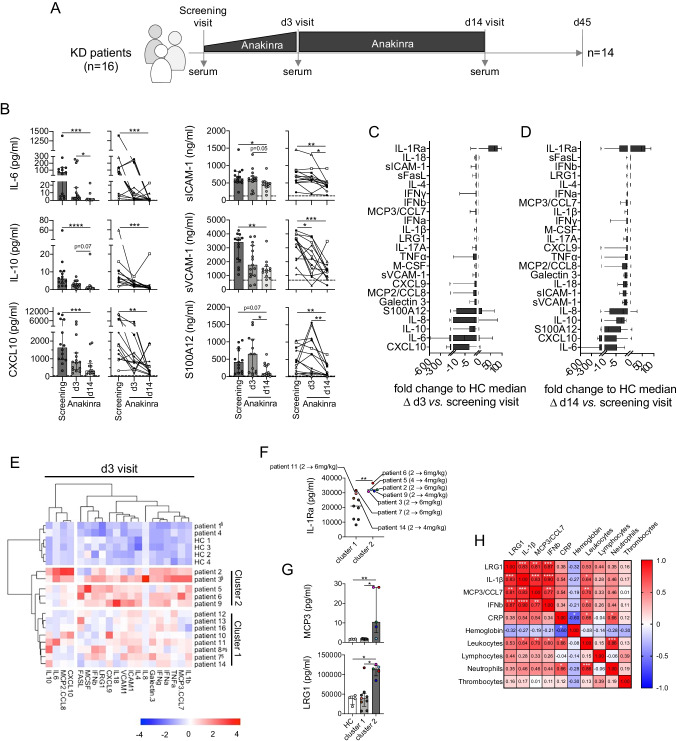
Selected serum biomarkers are most affected by IL-1R blockade and associate with the need to escalate anakinra dosage. **A** Schematic representation of the KAWAKINRA study protocol indicating time points of serum sampling. **B** Serum biomarker levels which revealed significant reduction in circulating levels upon anakinra treatment in both grouped (left panels) and individual analyses (right panels). Dashed lines indicate respective pediatric health control (*n* = 4) medians. Data were analyzed by Kruskal–Wallis followed by Dunn’s multiple comparison test (right panels) or Friedmann’s multiple comparison test for paired samples (left panels). **C**, **D** Anakinra-induced fold change to HC median on MFI level at days 3 (**C**) and 14 (**D**). **E** Ward’s unsupervised hierarchical clustering of serum biomarker levels (excluding IL1-Ra) quantified at d3 visit. Color coding indicates *Z* score, and clusters are annotated according to Fig. 1D. **F** IL-1Ra (anakinra) serum levels in study participants at d3. Color coding of patients informs on the need to escalate anakinra dosage. **G** Serum biomarker concentrations with significant differences between patients’ clusters. Data were analyzed by Kruskal–Wallis followed by Dunn’s multiple comparison test. **H** Correlation matrix of selected serum biomarker expression levels (MFI) and inflammatory parameter concentrations or cell counts. Color coding reflects Spearman correlation coefficient, and white stars indicate significance level of associations. * = *p* < 0.05; ** = *p* < 0.01; *** = *p* < 0.001; **** = *p* < 0.0001. ^§^Patients receiving steroids alongside with anakinra; ^#^patient 8 received only a single over-dosed injection of anakinra

Most serum marker levels quantified in samples collected at screening visit, following 3 days of anakinra treatment (d3) and at day 14 (d14, end of anakinra treatment; Fig. 2A), rapidly declined in course of anakinra treatment (Fig. 2B-D). Reduction upon blockade of IL-1 signaling was most pronounced for IL-6, IL-10, CXCL10, sICAM-1, sVCAM-1, and S100A12, at both cumulative (Fig. 2B, left panels) and individual patient follow-up levels (Fig. 2B, right panels). Compared to screening visit levels, decline following 3 and 14 days of anakinra was most pronounced for CXCL10 (median Δ to d3 =  − 14.64; median Δ to d14 =  − 21.82), IL-6 (− 5.25; − 42.75), S100A12 (− 0.59; − 8.38), IL-10 (− 1.48; − 2.80), and IL-8 (− 0.88; − 2.0). Increase in IL-1Ra levels on days 3 and 14 compared to screening visit reflect anakinra treatment (median Δ to d3 = 68.79; median Δ to d14 = 46.82; Fig. 2C, D and S2A).

Following 3 days of anakinra treatment, unsupervised hierarchical clustering separated the patient cohort into two main groups (Fig. 2E), which overlapped strongly with the biomarker-induced clustering at the screening visit (Fig. 1D) and reflected persistent systemic inflammatory activity among patients in cluster two over those in cluster one as well as patients 1 and 4 already associating with healthy controls. IL-1Ra levels quantified on study day 3 were not considered in this analysis, as—in contrast to measurements at screening visit—these levels would rather reflect anakinra than endogenous IL-1Ra concentrations. When analyzing peripheral IL-1Ra levels separately, we observed significantly higher levels of IL-1Ra in patients in cluster 2 than in cluster 1 (Fig. 2F). Four out of five patients in cluster 2 versus 3 out of nine patients in cluster 1 (including patients 1 and 4) required anakinra dose escalation (Fig. 2F). Patient 8 who only received a single but falsely too high dose of anakinra was excluded from this as well as subsequent analysis.

When further analyzing individual biomarker levels, we again observed absolute levels of LRG1 and MCP3 as well as MFI levels of IL-1β and IFN-β to best separate patients in cluster 2 from cluster 1 and healthy controls (Fig. 2G, S3A). Other quantified blood biomarkers were likewise elevated among individuals in cluster 2 but expression differences compare to cluster 1 remained far below significance level (Figure S3B). Clinical routine markers of inflammation indicated increased inflammatory activity among patients in cluster 2 only be trend (leukocyte/PMN/lymphocyte counts) or not at all (hemoglobin, CRP; Figure S3C).

Multiple correlation analyses of cluster differentiating serum markers (LRG1, MCP3, IL-1β, IFN-β), inflammatory parameters, and cell counts indicated marked positive associations among blood biomarkers as well as leukocyte counts (Fig. 2H).

Performing unsupervised hierarchical clustering analyses at the end of the anakinra treatment period (d14; Figure S4), biomarker-driven grouping of patients as observed at screening and d3 visit was almost lost.

### Prediction of Anakinra Treatment Regiment

**Fig. 3:**
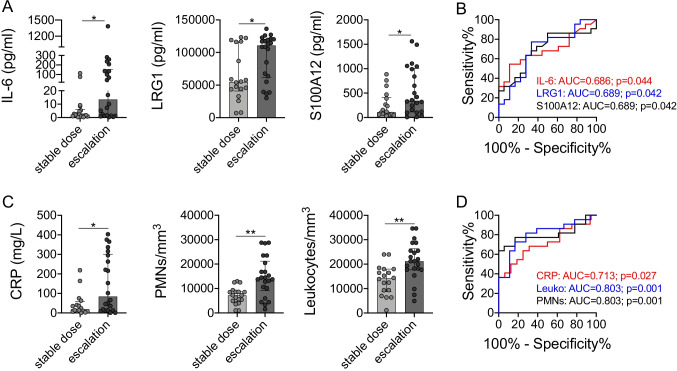
Collective serum biomarker and inflammatory parameter levels stratified by study outcome. **A** Collective serum biomarkers or **C** inflammatory parameters revealing statistically significant differences in levels when grouped according to the necessity to escalate anakinra dosage throughout the study. **B**, **D** Receiver operating curve analyses of respective biomarkers (**B**) or inflammatory parameters (**D**) associating with the necessity to escalate anakinra dosage. Data were analyzed by Kruskal–Wallis followed by Dunn’s multiple comparison (**A**) or Mann–Whitney *U* test (**C**). * = *p* < 0.05; ** = *p* < 0.01

Apart from unsupervised biomarker-driven analyses of the study cohort, we also stratified serum biomarker and inflammatory parameter data based on study outcome. We observed that over all samples, absolute serum concentrations of IL-6, LRG1, and S100A12 were significantly elevated among patients requiring anakinra dose escalation (Fig. 3A, B). Similarly, CRP concentrations as well as blood PMN and leukocyte counts were higher among this subgroup of IVIG-resistant KD patients (Fig. 3C, D). Furthermore, we wondered if serum biomarker and/or inflammatory parameter levels prior to IL-1Ra treatment might already facilitate a prediction on whether a patient may require future dose adjustment of anakinra. In these analyses of serum marker levels at screening visit, we observed IL-6, LRG1, and S100A12 serum levels to indicate future dose escalation only by trend (Figure S5A, B), while CRP concentrations (AUC = 0.85, *p* = 0.028, 95% CI = 0.64–1.0; cut-off > 45.5 mg/L, 100% Sens, 66.7% Spec) as well as blood PMN (AUC = 0.89, *p* = 0.013, 95% CI = 0.70–1.0; cut-off > 9730 cells/mm^3^, 88.9% Sens, 83.3% Spec) and leukocyte counts (AUC = 0.89, *p* = 0.014, 95% CI = 0.72–1.0; cut-off > 15,335 cells/mm^3^, 87.5% Sens, 83.3% Spec) in patients requiring future anakinra dose escalation were already significantly elevated at screening visit (Figure S5C, D).

### Association of LRG1 Expression with IL-1 Signaling

**Fig. 4:**
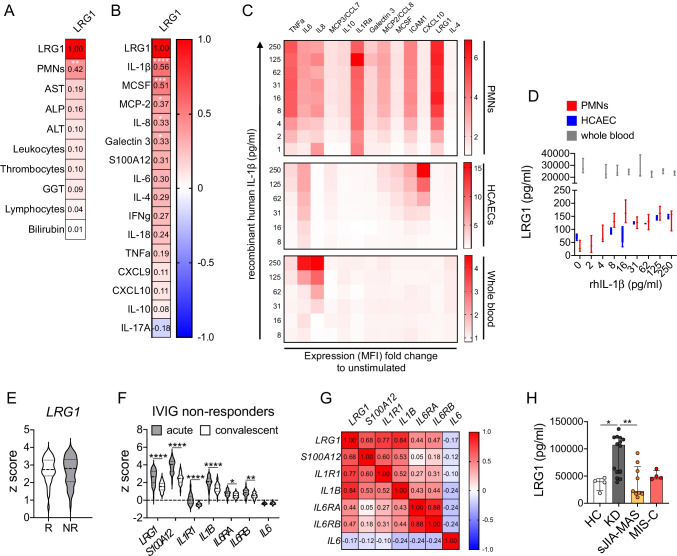
LRG1 expression associates with IL-1 signaling and is KD-specific. **A**, **B** Among all molecules with an immunological signaling capability (cytokines, chemokines) assessed in KAWAKINRA study samples, LRG-1 serum levels associate particularly with IL-1β. **C**, **D** Median MFI expression levels (**C**) or absolute concentrations (**D**) of indicated markers into culture supernatants of human neutrophils (*n* = 4 independent donors), human coronary artery endothelial cells (HCAECs, 4 independent experiments), or fresh human whole blood (4 independent donors) following stimulation (4 h) with indicated concentrations of recombinant human IL-1b. **E** *LRG1* expression (*Z* score of log10-transformed raw data, GSE63881) in IVIG responders (R, *n* = 220) and non-responders (NR, *n* = 121). **F** Expression of indicated genes (*Z* score of log10-transformed raw data, GSE63881) in paired samples of IVIG-resistant KD patients during acute and convalescent phase. **G** Pearson correlation matrix of indicated gene expression in IVIG-resistant patients during acute phase. **H** LRG1 levels in sera of healthy pediatric controls (*n* = 4), KD (KAWAKINRA screening visit data, *n* = 15), sJIA-associated MAS (*n* = 9), and MIS-C patients (*n* = 4). Data were analyzed by students’ unpaired (**E**) or ordinary one-way ANOVA followed by Sidak’s multiple comparison test (**F**). * = *p* < 0.05; ** = *p* < 0.01; *** = *p* < 0.001; **** = *p* < 0.0001

Among all serum biomarker analyses in IVIG-resistant KD patients, LRG1 stood out most consistently as overexpressed in a subgroup of patients with enhanced inflammatory activity and largely associated with the need to increase respective anakinra dosage over the course of treatment. LRG1 belongs to the leucine-rich repeat (LRR) protein family and is expressed by many cell types including endothelial cells, but is mainly produced by hepatocytes and myeloid cells, particularly granulocytes [22]. When analyzing association of collective LRG1 serum levels (all study visits) with circulating neutrophil (PMN), leukocyte, thrombocyte, and lymphocyte counts as well as markers indicating hepatic stress (transferases), the only significant correlation we observed was between LRG1 and peripheral neutrophil levels (Fig. 4A). Furthermore, we performed a collective correlation analysis of LRG1 with all potentially proinflammatory markers assessed in our study. From these analyses, we excluded type 1 interferons and MCP-3, due to their generally low expression in our assays, as well as sVCAM-1, sICAM-1, and sFasL as molecules less involved in direct inflammatory signaling. IL-1Ra was excluded as quantified levels beyond screening visit rather reflect anakinra than endogenous expression. Among all included inflammation modulating cytokines, chemokines, and DAMPs, this multiple correlation analysis indicated LRG1 to specifically associate with IL-1β concentrations throughout (Fig. 4B).

In order to further investigate a link between IL-1β signaling and LRG1 expression, we stimulated primary human coronary artery endothelial cells (HCAECs) with previously demonstrated high sensitivity to IL-1β signaling [10] healthy donor whole blood, and isolated neutrophils as among the main LRG1 producers [22] with recombinant human IL-1β. Here, we observed concentration-dependent LRG1 release from particularly isolated human neutrophils, which were also responsive to recombinant human IL-1β on the level of other molecules (i.e., TNF, IL-6, IL-8, IL-1Ra, sICAM-1; Fig. 4C, D). Compared to neutrophils, IL-1β stimulation induced only minor LRG1 release from HCAECs. Without stimulation, basal LRG1 levels in whole blood were already markedly elevated over those quantified in both PMN and HCAEC supernatants and in range with those previously observed in healthy control sera and could not be increased any further by stimulation with recombinant human IL-1β (Fig. 4C, D).

Next, we aimed at validating our blood biomarker data in a different KD cohort and on gene rather than protein expression level. Therefore, we used already available blood transcriptional profile data of 146 KD patients in course of IVIG treatment (GSE63881), which had previously highlighted a prominent role of the IL-1β pathway in disease [7]. In this retrospective analysis of *Z* score on normalized data based on log10-transformation, we observed *LRG1* expression not to differ between IVIG responders and non-responders (Fig. 4E). In line with our data obtained from the KAWAKINRA study cohort, we further compared expression levels of *LRG1* in acute and convalescent phase with those of molecules involved in IL-1 signaling (*IL1R1*, *IL1B*), not only *S100A12* as neutrophil marker but also *IL6* as top de-regulated in KAWAKINRA, as well as *IL6RA* and *IL6RB*. We found all significantly reduced upon convalescence (Fig. 4F), except for *IL6*, which contrasts our serum marker data (Figs. 1B, 2B). Furthermore, we observed strongest correlations between *LRG1* and *IL1B* (*r* = 0.84, *p* = 1.47⋅10^−17^), *IL1R1* (*r* = 0.77, *p* = 6.44⋅10^−13^), and *S100A12* (*r* = 0.68, *p* = 2.1⋅10^−9^; Fig. 4G).

Finally, we compared LRG1 serum levels in IVIG-resistant KD patients with concentrations quantified in MAS as a hyperinflammatory condition, which can also complicate KD [5]. Respective data were obtained from one patient enrolled in KAWAKINRA but retrospectively diagnosed as sJIA-MAS (patient 15) as well as patients previously reported in other context [18] (Table 1). Furthermore, we included LRG1 levels in MIS-C in this comparison, as this condition is thought to share clinical features with KD [23, 24] (Table 1). Collectively, we observed elevated LRG1 levels to separate the investigated IVIG-resistant KD patients (particularly those with high inflammatory activity) from both sJIA-MAS and MIS-C (Fig. 4H).

## Discussion

In the investigated IVIG-resistant KD patients, inflammation upon study entry is hallmarked by over-expression of innate immune mediators, particularly IL-6, CXCL10, S100A12, and IL-1Ra. Subsequent treatment with anakinra decreased those most significantly, albeit almost all investigated blood biomarkers indicative of innate and/or adaptive immune mechanisms or (immune) cell activation indicated declining inflammation following IL-1R blockade. Throughout, increased serum LRG1 levels were associated with a subgroup of patients with elevated inflammatory activity, requiring escalation of anakinra dosage and separated those from (hyper)inflammatory conditions such as in sJIA-associated MAS or MIS-C. LRG1 expression on both protein and gene levels tightly associated with IL-1β levels and signaling as well as blood neutrophil counts, and IL-1 signaling in human neutrophils induced concentration-dependent LRG1 release.

Following IVIG unresponsiveness, we observed elevation of serum markers rather attributable to innate immunity, which reflects previous observations [25]. Compared to healthy controls, particularly serum levels of IL-6 were strongly upregulated in our study cohort. IL-6 levels have been previously reported as among the most responsive to successful IVIG treatment [26, 27], while persistent elevated levels can indicate refractory disease [27]. Following anakinra treatment of IVIG non-responders, those as well as several others of the investigated marker levels normalized. This supports the proposed central role of IL-1 in KD pathophysiology and is in line with the previously reported clinical improvements observed in the study cohort upon anakinra treatment [17].

Albeit approximately 80% of KAWAKINRA study patients became afebrile within 48 h following the last anakinra dose escalation and disease activity measures such as physician’s and parents’ evaluations as well as CRP levels improved in almost all cases [17], in a subgroup of patients, serum levels of inflammatory markers and LRG1 in particular remained elevated throughout. Unbiased biomarker-driven hierarchical clustering analyses prior to and following 3 days of anakinra treatment suggested particularly elevated LRG1 concentrations to associate with persistent inflammation as reflected by respective clinical inflammatory parameters as well as the necessity to escalate anakinra dosage.

LRG1 behaves like an acute phase protein as it is mainly produced by hepatocytes following inflammatory stimulation. Beyond, it can be expressed by many cell types but particularly granulocytes, where it is suggested to have a role in cell differentiation [22, 28]. By modulating TGF-β signaling, LRG1 is thought to be implicated in endothelial activation, vascular dysfunction, neovascularization, and cardiac re-modeling via fibroblast activation and cardiac fibrosis [29, 30]. Importantly, genetic variation in the TGF-β pathway have been demonstrated to influence KD susceptibility, disease outcome, and response to therapy and are thought to support the importance of this pathway in KD pathogenesis [31].

In context with KD, LRG1 has already been reported as elevated in sera from acute phase KD patients compared with individuals in the recovery phase and healthy controls [32] and double positivity for both LRG1 and angiotensin has been suggested as biomarker for differentiating incomplete KD from non-KD patients [33]. Furthermore, LRG1 has been found selectively upregulated in serum exosomes isolated from KD patients with CAAs [34]. As also demonstrated by our data, this collectively suggests LRG1 expression and serum levels to reflect increased inflammatory activity in specifically KD.

Importantly, LRG1 expression on both protein and gene levels associated with IL-1β signaling as well as blood neutrophil counts, and IL-1β stimulation of particularly human neutrophils induced concentration-dependent LRG1 release. As already demonstrated elsewhere [35], cells in whole blood remained comparably unresponsive to recombinant IL-1β, likely due to blockade or decoy of IL-1β by endogenous IL-1Ra and IL-1R2, respectively. Studies in context with other inflammatory conditions such as on adult-onset Still’s disease (AOSD) [36] or Crohn’s disease [37] similarly suggest an association of IL-1β and LRG1 expression. In contrast, studies linking LRG1 with IL-6 or TNF signaling report rather contradictory results [36, 38, 39], which are thus partly in line [37, 38] with the weak associations observed among our KD data.

Albeit LRG1 levels have been reported elevated in both KD and sJIA [38], we observed selective over-expression of LRG1 in KD when compared to sJIA-associated MAS. Similar to MAS, LRG1 serum concentrations in MIS-C, which has been reported to share overlapping clinical features with KD [23, 24], are not elevated compared to healthy controls and thus both sJIA-MAS and MIS-C clearly separate from at least a subpopulation of the investigated KD patients. Compared to KD, we observed less or no association of LRG1 with circulating IL-1β in both sJIA-MAS and MIS-C, which may point towards a different inflammatory pathophysiology with yet unclear involvement of IL-1 signaling [23, 40].

While, collectively, our data may raise speculations for a previously undiscovered patho-mechanistic involvement of an IL-1β-LRG1 axis as driver of cardiac re-modeling in KD, it still requires appropriate models and approaches to test for this. In a substantially larger patient cohort, we are now validating whether LRG1 serum levels can indeed inform on KD disease course and/or differentiate KD and MIS-C. Similarly, therapeutic targeting of LRG1 is being tested. In other disease context, therapeutic LRG1 inhibition has already been suggested to restore normal vascular function [41, 42]. In the light of recent findings on depletion of bioactive IL-1β-loaded neutrophils as a central therapeutic mechanism in KD [43], one may further speculate that in IVIG-resistant KD particularly those cells may escape this depletion, which may be supported by our data on the prevalence of an IL-1β-LRG1-neutrophil axis in the present study population. In the biomarker context of the present study, IL-6, S100A12, and LRG1 could offer some predictive power on the response of IVIG-resistant KD patients to therapeutic IL-1 blockade; however, data on the predictive potential of these markers already prior to start of anakinra therapy were inconclusive due to lack of statistical power within the small study population. Nevertheless, conventional markers of inflammation such as peripheral leukocyte and neutrophil counts as well as CRP levels were indeed able to indicate a future anakinra dose adjustment despite the small cohort size.

## Supplementary Information

Below is the link to the electronic supplementary material.

Supplementary file1 (DOCX 2069 KB)

## Data Availability

The datasets generated during and/or analyzed in course of this study as well as study-specific analysis reagents are available from the corresponding author upon reasonable request.

## Code Availability

No specific codes have been used in course of data analyses. All applied R codes are standard scripts and can be obtained from the corresponding author upon reasonable request.
