# Supplementary material for: An Immunological Axis Involving Interleukin 1β and Leucine-Rich-α2-Glycoprotein Reflects Therapeutic Response of Children with Kawasaki Disease: Implications from the KAWAKINRA Trial
Source: J Clin Immunol. 2022 Jun 14;42(6):1330–41. doi: 10.1007/s10875-022-01301-w (PMC9537216; doi:10.1007/s10875-022-01301-w)
Supplement: Supplementary file 1 — Supplementary file1 (DOCX 2069 KB) [file 10875_2022_1301_MOESM1_ESM.docx]

Supplemental methods & figures

An immunological axis involving interleukin 1β and leucine-rich-α2-glycoprotein reflects therapeutic response of children with Kawasaki disease: implications from the KAWAKINRA trial

Christoph Kessel^a^, Isabelle Koné-Paut^b^, Stéphanie Tellier^c^, Alexandre Belot^d^, Katja Masjosthusmann^e^, Helmut Wittkowski^a^, Sabrina Fuehner^a^, Linda Rossi-Semerano^b^, Perrine Dusser^b^, Isabelle Marie^b^, Nadja Boukhedouni^f^, Helène Agostini^f^, Céline Piedvache^f^ and Dirk Foell^a^

^a^Department of Pediatric Rheumatology and Immunology, University Children’s Hospital Muenster, Muenster, Germany

^b^Division of Pediatric Rheumatology and CEREMAIA, Bicêtre Hospital, APHP, University of Paris Saclay, Le Kremlin-Bicêtre, France

^c^Department of Pediatrics, Divisions of Nephrology, Rheumatology and Internal Medicine, University of Toulouse, Toulouse, France

^d^Departments of Pediatrics, Division of Rheumatology, Dermatology and Nephrology, University of Lyon, Lyon, France

^e^Department of General Pediatrics, University Children's Hospital Muenster, Muenster, Germany

^f^APHP, Paris Saclay, Clinical Research Unit Paris-Saclay, Bicêtre Hospital, Le Kremlin-Bicêtre, France

**Corresponding author:** Dr. Christoph Kessel, Department of Pediatric Rheumatology and Immunology, University Children’s Hospital Muenster, Domagkstr. 3, 48149 Muenster, Germany; Email: [christoph.kessel@uni-muenster.de](mailto:christoph.kessel@uni-muenster.de); Phone: +49-251-83-58176; Fax: +49-251-83-58174

**Cell stimulations**

Human heparinized whole blood was collected in respective vacutainers. Polymorphonuclear leukocytes (PMNs) were isolated from human whole blood using the EasySep™ Direct Human Neutrophil Isolation Kit (Stemcell technologies, Cologne, Germany) according to the manufacturer’s instructions. 150μl of fresh blood or 25 x 10^4^ PMNs/200μl culture medium (complete RPMI) were stimulated with indicated concentrations of recombinant human IL-1β (R&D Systems, Minneapolis, MN, USA). Following 4h of stimulation supernatants were separated from whole blood components or PMNs by centrifugation (10’, 2000rpm) and stored at -20°C until analysis.

Human coronary artery endothelial cells (HCAECs) were purchased from PeloBiotech (Planegg, Germany) and were cultured and stimulated as described previously (1). Briefly, cells in microvascular endothelial cell growth medium (MEM, PeloBiotech) supplemented according to the manufacturer’s instructions were seeded in gelatin-coated (0.1%, Sigma-Aldrich, Munich, Germany) 12-well tissue culture plates (2 x 10^4^ cells/well in 2 ml complete MEM) and were stimulated with recombinant human IL-1β (R&D Systems) at 80-90% confluence. Following 18h of stimulation, supernatants were harvested and stored at -20°C until analysis.

**Quantification of single or multiple markers in sera and culture supernatants**

Reagents for multiplexed quantification of IL-1β, IL-1Ra, IL-4, IL-6, IL-8, IL-10, IL-17A, IL-18, TNFα, IFNα, IFNβ, IFN-γ, MCP2 (CCL8), MCP3 (CCL7), CXCL9, CXCL10, MCSF, LRG1, sFasL, ICAM-1, VCAM-1 and Galectin-3 were purchased from R&D Systems (Minneapolis, OH, USA). Reagents and sera or cell culture supernatants were prepared according to the manufacturer’s instructions (R&D Systems). KAWAKINRA study samples as well as healthy controls were run on a single 96-well Luminex plate. Data acquisition and analysis was performed on a MAGPIX instrument (Merck Millipore, Darmstadt, Germany) using xPONENT v4.2 software (Luminex). Concentrations of S100A12 in patients’ sera were quantified by sandwich ELISA using in-house monoclonal antibodies (1).

**Figure S1. Serum biomarker levels at screening visit.** (**A**) Ward’s unsupervised hierarchical clustering of serum biomarker levels quantified at screening visit, including patient 15 which was retrospectively diagnosed as sJIA-MAS. Color coding indicates Z-score. (**B**) Serum biomarkers which support patient clustering at screening visit on MFI but not absolute concentration level. (**C**, **D**) Serum biomarker levels (**C**) and blood cell counts (**D**) which do not significantly support patient clustering at screening visit. Data were analyzed by Kruskal-Wallis followed by Dunn’s multiple comparison (**B**, **C**) or Mann-Whitney U test test (**D**). * =p<0.05; ** =p<0.01.

**Figure S2. IL-Ra and inflammatory parameter levels in course of anakinra.** Data show cumulative (left panels) or individual analyses (right panels). Dashed lines indicate respective pediatric healthy control (n=4) medians. Data were analyzed by Kruskal-Wallis followed by Dunn’s multiple comparison test (right panels) or Friedmann’s multiple comparison test for paired samples (left panels). * =p<0.05; ** =p<0.01; *** =p<0.001

**Figure S3. Serum biomarker levels** **at d3 visit.** (**A**) Serum biomarkers which support patient clustering at screening visit on MFI but not absolute concentration level. (**B**, **C**) Serum biomarker levels (**B**) and blood cell counts (**C**) which do not significantly support patient clustering at screening visit. Color coding of patients informs on the need to escalate anakinra dosage according to figure 2F. Data were analyzed by Kruskal-Wallis followed by Dunn’s multiple comparison (**A**, **B**) or Mann-Whitney U test (**C**). * =p<0.05; ** =p<0.01.

**Figure S4. Serum biomarker levels** **at d14 visit.** (**A**) Ward’s unsupervised hierarchical clustering of serum biomarker levels (excluding IL1-Ra) quantified at d14 visit. Color coding indicates Z-score.

**Figure S5. Predicition of anakinra dose adjustment in IVIG-resistant KD.** (A, C) Selected serum biomarker (A) or inflammatory parameter (C) levels at screening visit, grouped according to the necessity to escalate anakinra dosage during the first 3 days of treatment. Patients in red indicate those who previously received glucocorticosteroids. (B, D) Receiver operating curve analyses of selected biomarkers (B) or inflammatory parameters (D) to predict the necessity to escalate anakinra dosage. Data were analyzed by Kruskal-Wallis followed by Dunn’s multiple comparison (**A**) or Mann-Whitney U test (**C**). * =p<0.05; ** =p<0.01.

References

1. Armaroli G, Verweyen E, Pretzer C, Kessel K, Hirono K, Ichida F, et al. Monocyte-Derived Interleukin-1beta As the Driver of S100A12-Induced Sterile Inflammatory Activation of Human Coronary Artery Endothelial Cells: Implications for the Pathogenesis of Kawasaki Disease. Arthritis Rheumatol. 2019;71(5):792-804.
